# Hotspot mutant p53-R273H inhibits KLF6 expression to promote cell migration and tumor metastasis

**DOI:** 10.1038/s41419-020-02814-1

**Published:** 2020-07-30

**Authors:** Shengnan Sun, Hu Chen, Lijuan Sun, Miao Wang, Xianqiang Wu, Zhi-Xiong Jim Xiao

**Affiliations:** Center of Growth, Metabolism and Aging, Key Laboratory of Bio-Resource and Eco-Environment, Ministry of Education, College of Life Sciences, Sichuan University, Chengdu, 610064 China

**Keywords:** Epithelial-mesenchymal transition, Oncogenesis

## Abstract

Hotspot p53 mutant proteins often gain novel functions in promoting tumor metastases. However, the molecular mechanisms by which mutant p53 exerts gain-of-function in cancer are not totally understood. In this study, we demonstrate that hotspot mutant p53, p53-R273H, promotes cell scattering growth and migration via inhibiting the expression of Krupple-like factor 6 (KLF6), a Zinc finger transcription factor and a documented tumor suppressor. Restoration of KLF6 increases the expression of E-cadherin downregulated by p53-R273H and inhibits p53-R273H-induced cell migration and tumor metastasis. Further, p53-R273H reduces KLF6 transcription by upregulating EGFR expression which in turn activates AKT–FOXO1 axis. Pharmacological inhibitor of AKT, MK2206, rescues KLF6 expression and suppresses p53-R273H-induced cell migration. Clinical analyses reveal that KLF6 expression is decreased in human breast cancer specimens harboring p53 mutations, and negatively correlated with EGFR expression in human breast cancer. In addition, low expression of KLF6 is associated with poor overall survival (OS) and relapse-free survival (RFS) in p53 mutated human breast cancer patients. Together, these results reveal an important role for EGFR–AKT–FOXO1–KLF6–E-cadherin axis in mutant p53-induced cell migration and tumor metastasis.

## Introduction

p53 is a critical tumor suppressor, which transactivates multiple target genes involved in controlling cell cycle arrest, apoptosis, senescence, DNA damage repair, and cell metabolism^1^. It has been documented that more than 50% human tumors bear p53 gene mutations, of which missense mutations account for over 70% of all p53 mutants^2^. Moreover, about 80% of missense mutations reside in central DNA-binding domain (DBD) of p53 protein, with several “hotspot” mutations including R175, R248, and R273, which often exerted a dominant-negative activity by interacting with wild-type p53^3^. While these hotspot mutations lost transactivating activity and tumor suppressor functions, they often gain new functions in promoting tumor initiation, development, and metastasis^4^, as exemplified that mice harboring mutants p53 develop more invasive and metastatic tumors than p53-null mice^5^.

E-cadherin, encoded by CDH1 and expressed mainly in epithelial cells, is a single transmembrane protein interacting with neighbor E-cadherin molecules expressed on adjacent cells that are involved in formation of epithelial adherent junctions^6^. It has been reported that the cytoplasmic domain of E-cadherin protein interacts with numbers of proteins, including α-catenin, β-catenin, and p120-catenin, which mediate contacts between E-cadherin and actin cytoskeleton, thereby playing a pivotal role in cell–cell adhesion and suppressing cell migration and invasion^7^. Downregulation of E-cadherin is essential for epithelial–mesenchymal transition (EMT), a unique morphogenetic change during embryonic development and tumor metastasis^8^. E-cadherin expression is tightly controlled at genetic, epigenetic, transcriptional, and posttranslational levels during cancer development^9^.

The Krupple-like factor 6 (KLF6) is an evolutionally conserved and ubiquitously expressed transcription factor in mammals^10^. KLF6 gene is located on chromosome 10 of human genome and has three splice variants, SV1, SV2, and SV3^11,12^. The KLF6 protein consists of an acidic domain exerting transcriptional activity, a Ser/Thr-rich domain and a DBD which contains three Cys_2_-His_2_ zinc fingers structure^12^. Inactivation of KLF6 has been frequently found in most human cancers, including lung, hepatocellular, colorectal, prostate, gastric, nasopharyngeal, astrocytic glioma, and ovarian cancer^13^. It has been reported that KLF6 suppresses cancer cell growth through transactivation of p21 expression in a p53-independent manner^14^. KLF6 directly activates E-cadherin transcription to inhibit tumor invasion and metastasis^13^. Besides, KLF6 also suppresses cell migration and tumor metastasis in an E-cadherin-independent manner. For instance, KLF6 represses hepatocellular carcinoma cell migration by suppressing the expression of VAV3, a known activator of the RAC1 small GTPase^15^. In addition, KLF6 also suppresses cancer cell metastasis via repressing the transcription of E2F1^16^ or MMP9^17^.

In this study, we demonstrate that p53-R273H promotes tumor metastasis by downregulation of KLF6 gene transcription via the EGFR-AKT-FOXO1 pathway. p53-R273H-induced tumor metastasis is effectively reversed by restoration of KLF6 expression. Clinical analyses show that KLF6 expression is significantly reduced in p53 mutant human breast cancer, and is negatively correlated with EGFR expression. And low expression of KLF6 is associated with poor overall survival (OS) and relapse-free survival (RFS) in p53 mutated human breast cancer patients.

## Results

### p53-R273H promotes cell migration via downregulation of KLF6 and E-cadherin expression

**Fig. 1:**
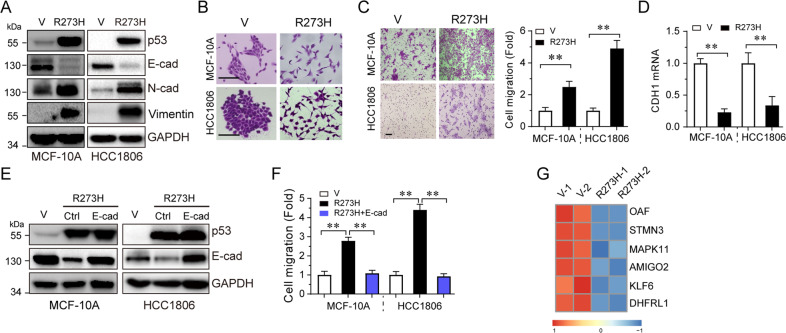
p53-R273H promotes cell migration via downregulation of E-cadherin. Stable MCF-10A or HCC1806 cells expressing vector control (V) or p53-R273H (R273H) were subjected to western blot analyses for p53 and EMT markers as indicated (**a**), colony formation (**b**), transwell (**c**), or qPCR analyses (**d**). Stable MCF-10A or HCC1806 cells expressing p53-R273H were infected with lentivirus encoding E-cadherin, then subjected to western blot analyses (**e**) or transwell analyses (**f**). Scale bar = 100 μm. **P < 0.01. Results are presented as means ± SD from three independent experiments in triplicates. **g** The p53-R273H-suppressed genes coupled with p53-R273H ChIP-seq analyses, as described in the “Materials and Methods”, were represented in the heat map.

p53 is the most frequently mutated gene in human cancers. More than half of all types of human tumors have p53 mutations or deletions. It has been well documented that mutant p53 proteins not only lost the tumor suppressing function or act to inhibit wild-type p53 function in a dominant-negative fashion, they often gain new functions to promote tumor development and metastasis. However, how mutant p53 promotes metastasis is not totally understood. To explore the molecule mechanisms by which mutant p53 promotes cell migration, we constructed stable MCF-10A (p53-wild-type) or HCC1806 (p53-null) cells expressing p53-R273H and examined the effect of p53-R273H on the expression of known EMT markers. As shown in Fig. 1a, expression of p53-R273H led to upregulation of Vimentin and N-cadherin and downregulation of E-cadherin protein expression, concomitant with scattered cells growth (Fig. 1b) and cell migration (Fig. 1c). Notably, p53-R273H downregulated E-cadherin mRNA, suggesting that p53-R273H suppresses E-cadherin gene transcription (Fig. 1a, d). In addition, restoration of E-cadherin inhibited p53-R273H-induced cell migration (Fig. 1e, f). Together, these results indicate that p53-R273H induces cell migration via suppression of E-cadherin gene transcription, keeping in line with a previous report that p53-R273H downregulates E-cadherin expression and alters cell polarity via EMT^18^.

**Fig. 2:**
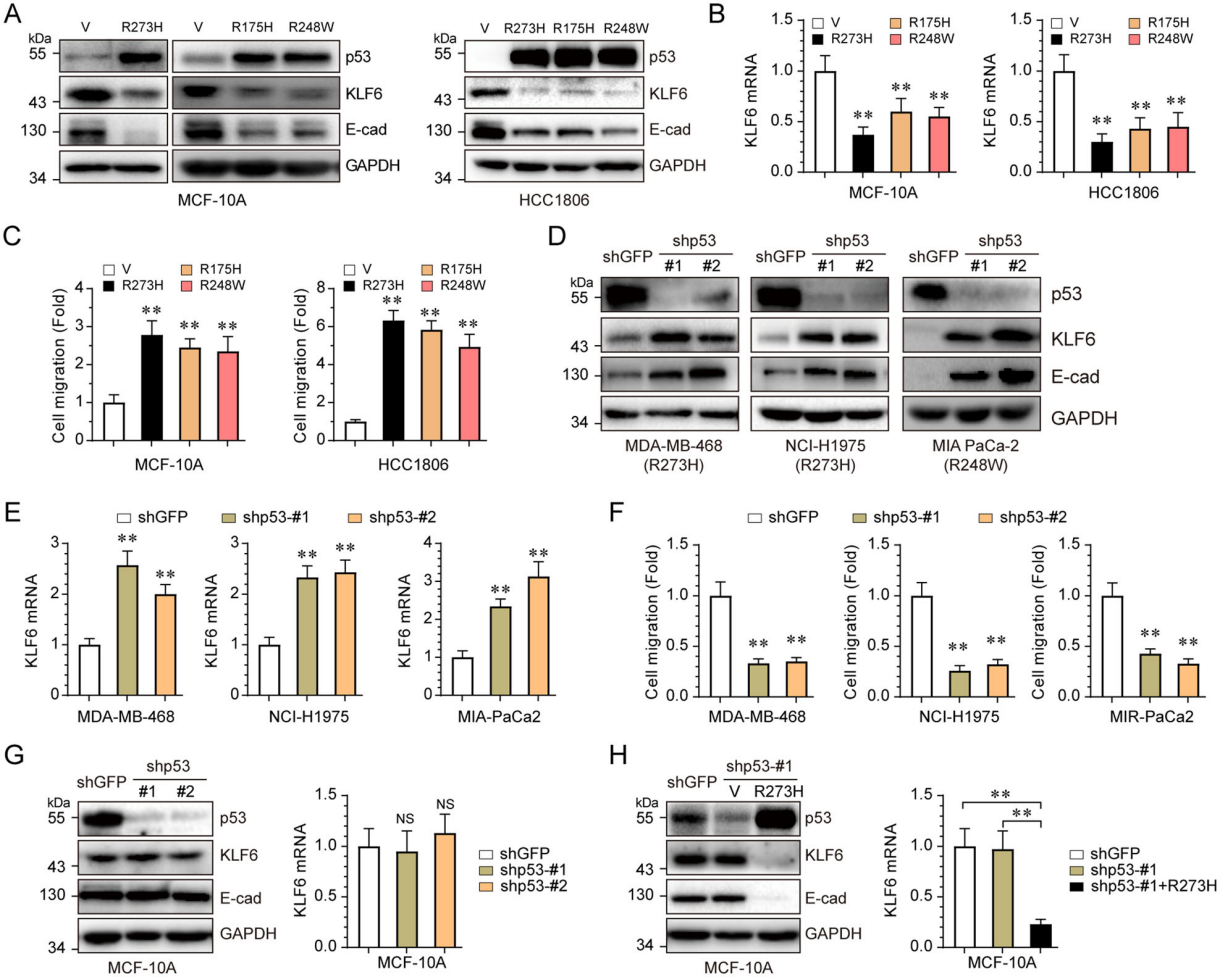
p53 hotspot mutants suppress KLF6 expression. Stable MCF-10A or HCC1806 cells expressing either p53-R273H, p53-R175H, or R248W were subjected to western blotting (**a**), qPCR (**b**), or transwell assays (**c**). MDA-MB-468, NCI-H1975, or MIA PaCa-2 cells were infected with lentivirus expressing either of two different shRNAs specific for p53 (shp53-#1 and shp53-#2), or a control shRNA (shGFP), followed by western blotting analyses (**d**), qPCR (**e**), or transwell assays (**f**). MCF-10A cells were infected with lentivirus expressing either of two different shRNAs specific for p53 (shp53-#1 and shp53-#2), or a control shRNA (shGFP), followed by western blotting or qPCR assays (**g**). MCF-10A stable cells expressing shp53-#1 (which targets the 3′ UTR of p53 mRNA) were infected with lentivirus encoding p53-R273H, prior to western blotting or qPCR assays (**g**). **P < 0.01, NS indicated no significance. Results are presented as means ± SD from three independent experiments in triplicates.

To further define the mechanism with which mutant p53 suppresses E-cadherin expression, we employed integrated analyses of gene expression profiles^19^ and ChIP-seq data^20^, in which p53-R273H acts as a transcriptional co-factor. As shown in Fig. 1g, we identified six genes that were downregulated by p53-R273H and that p53-R273H bound to their promoter regions. Notably, among these six genes, KLF6 is a documented tumor suppressor protein that transactivates E-cadherin gene expression^13^. We therefore hypothesized that p53-R273H downregulates E-cadherin expression via inhibition of KLF6. With this regard, we found that not only p53-R273H, but also other hotspot mutants, p53-R175H and p53-R248W, significantly inhibited KLF6 expression at both mRNA (Fig. 2a) and protein levels (Fig. 2b), and promoted cell migration (Fig. 2c). In addition, knockdown of endogenous mutant p53 in MDA-MB-468 (p53-R273H), NCI-H1975 (p53-R273H), and MIA PaCa-2 (p53-R248W) cells led to significant upregulation of the mRNA and protein levels of both KLF6 and E-cadherin (Fig. 2d, e), resulting in inhibition of cell migration (Fig. 2f). By sharp contrast, knockdown of endogenous wild-type p53 in MCF-10A cells had little effect on the expression of KLF6 and E-cadherin (Fig. 2g). Furthermore, ectopic expression of p53-R273H in p53-ablated MCF-10A cells dramatically inhibited KLF6 and E-cadherin expression (Fig. 2h). Together, these data indicate that p53 hotspot mutant proteins can suppress transcription of KLF6 and E-cadherin, concomitant with increased cell migration.

### KLF6 inhibits p53-R273H-induced cell migration and tumor metastasis

**Fig. 3:**
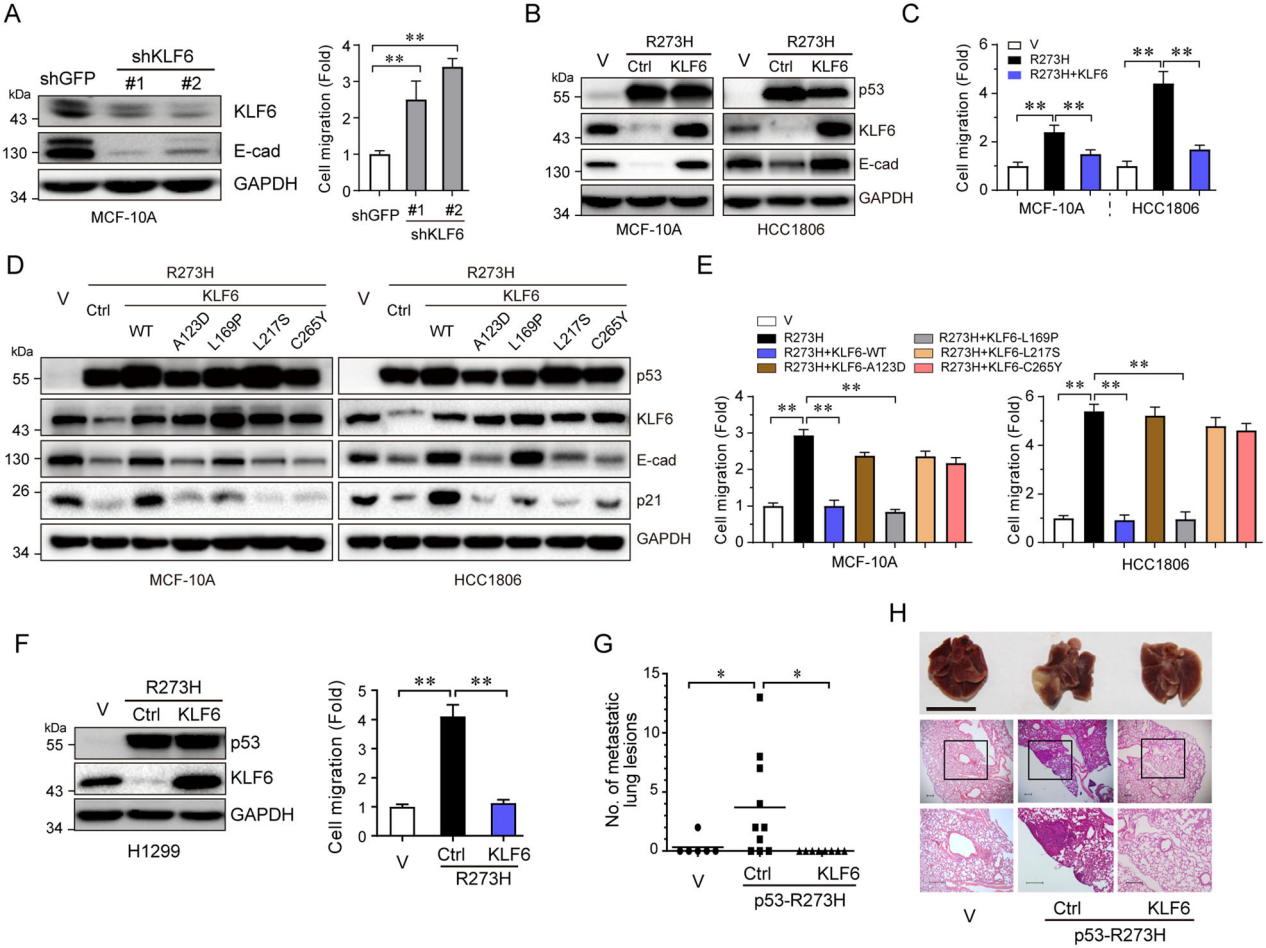
KLF6 suppresses p53-R273H-induced cell migration and tumor metastasis. MCF-10A cells were infected with lentivirus expressing either of two different shRNAs specific for KLF6 (shKLF6-#1 and shKLF6-2#), or a control shRNA (shGFP), followed by western blotting or transwell assays (**a**). Stable MCF-10A or HCC1806 cells expressing p53-R273H were infected with lentivirus encoding KLF6, then subjected to western blotting (**b**) and transwell assays (**c**). Stable MCF-10A and HCC1806 cells expressing p53-R273H were infected with lentivirus encoding KLF6 wild-type (WT) or mutations (A123D, L169P, L217S, and C265Y), then subjected to western blotting (**d**) or transwell assays (**e**). H1299 stable cells expressing p53-R273H were infected with lentivirus encoding KLF6, then subjected to western blotting and transwell assays (**f**). H1299 stable cells, expressing mutant p53-R273H or a vector control (V), were infected with lentivirus encoding KLF6 or a vector control (Ctrl), tail-vain injected into female nude mice (ten mice per group). Mice were observed daily and sacrificed after 50 days. Lungs were dissected and fixed, and were inspected for metastatic nodules on their surface (**g**). Lungs were fixed, embedded in paraffin, sectioned, and stained by H&E for histological analysis, Scale bars = 1 cm (**h**). *P < 0.05; **P < 0.01. Results are presented as means ± SD from three independent experiments in triplicates.

We next examined the effects of KLF6 on cell migration. Knockdown of KLF6 resulted in reduction of E-cadherin protein expression and increased cell migration (Fig. 3a). Restoration of KLF6 expression effectively recovered expression of E-cadherin suppressed by p53-R273H and significantly inhibited cell migration induced by p53-R273H in MCF-10A or HCC1806 cells (Fig. 3b, c). To investigate whether the transcriptional activity of KLF6 is required for inhibition of mutant p53-induced cell migration, we examined the effects of several KLF6 mutants defective in transcription activation^21^. As shown in Fig. 3d, e, expression of wild-type KLF6, but not its point mutation derivatives, including A123D, L169P, L217S, or C265Y, was able to upregulate p21, as expected^21^. Notably, neither A123D, L217S, or C265Y could stimulate E-cadherin expression, nor could they suppress p53-R273H-induced cell migration. Interestingly, although L169P was not unable to activate p21 expression, it could activate E-cadherin expression and suppress p53-R273H-induced cell migration, suggesting that KLF6 regulates the expression of its target genes differently.

Next, we investigated whether p53-R273H-mediated suppression of KLF6 can induce tumor metastasis. As shown in Fig. 3f, p53-R273H significantly reduced KLF6 expression in p53-null lung cancer H1299 cells. Restored expression of KLF6 dramatically inhibited p53-R273H-induced H1299 cell migration. Furthermore, we intravenously injected stable H1299 cells expressing p53-R273H with or without simultaneous KLF6 expression into the tail veins of recipient nude mice. As shown in Fig. 3g, h, mice bearing p53-R273H-expressing cells exhibited multiple metastatic nodules on the lung surfaces. However, simultaneous expression of KLF6 effectively suppressed the formation of metastatic nodules induced by p53-R273H. Together, these data indicate that KLF6 can suppress p53-R273H-induced H1299 cell migration and tumor metastasis.

### p53-R273H inhibits KLF6 transcription via EGFR–AKT–FOXO1 axis

**Fig. 4:**
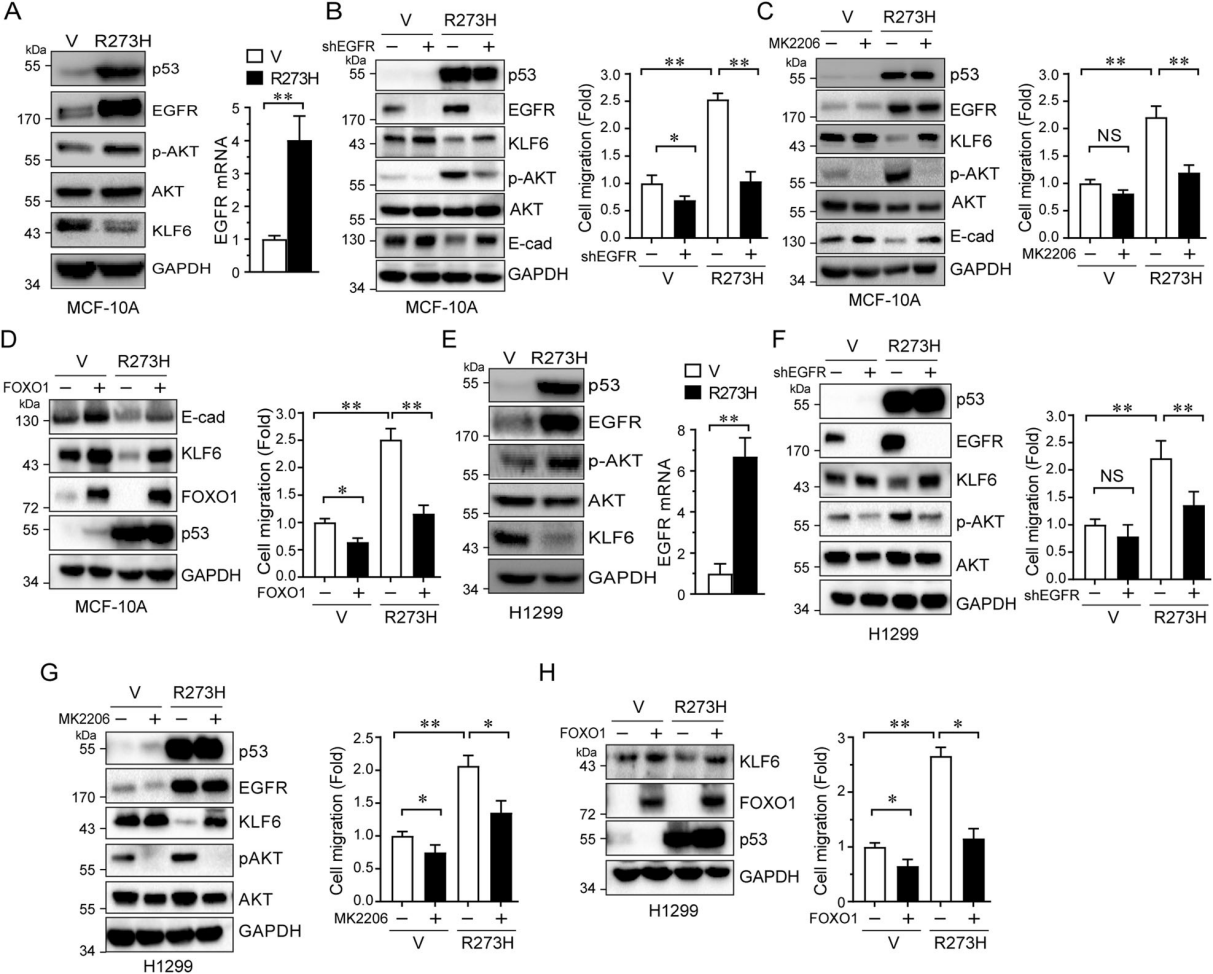
p53-R273H activates EGFR-AKT-FOXO1 pathway to inhibit KLF6 expression and promotes cancer cell migration. Stable MCF-10A (**a**–**d**), or H1299 (**e**–**h**) cells were used in the following experiments. Stable cells expressing p53-R273H were subjected to western blotting and qPCR analyses (**a**, **e**). Stable cells expressing p53-R273H were infected with lentivirus expressing shRNAs specific for shEGFR, then subjected to western blotting and transwell assays (**b**, **f**). Stable cells expressing p53-R273H were treated with MK2206 (5 μM) or DMSO for 24 h, then subjected to western blotting and transwell assays (**c**, **g**). Stable cells expressing p53-R273H were infected with lentivirus expressing FOXO1, then subjected to western blotting and transwell analyses (**d**, **h**). *p < 0.05; **p < 0.01; NS indicated no significance. Results are presented as means ± SD from three independent experiments in triplicates.

We then investigated the molecular basis with which p53-R273H downregulates KLF6 expression. As shown in Fig. 4a, p53-R273H significantly induced mRNA and protein levels of EGFR and AKT phosphorylation, concomitant with reduced KLF6 expression in MCF-10A cells. In addition, silencing of EGFR markedly restored KLF6 protein expression, concomitant with blockage of cell migration induced by p53-R273H (Fig. 4b). Furthermore, MK2206, a pharmacological inhibitor of AKT, significantly restored the expression of KLF6 and consequently inhibited p53-R273H-induced cell migration (Fig. 4c). Notably, p53-R273H significantly inhibited expression of FOXO1 (Fig. 4d); restoration of FOXO1 expression not only effectively restored expression of KLF6, but also completely inhibited cell migration induced by p53-R273H (Fig. 4d). Similar results were obtained in H1299 cells (Fig. 4e–h). Taken together, these data indicate that p53-R273H suppresses KLF6 expression via the EGFR–AKT–FOXO1 axis.

### Downregulation of KLF6 is correlated with breast cancer development

**Fig. 5:**
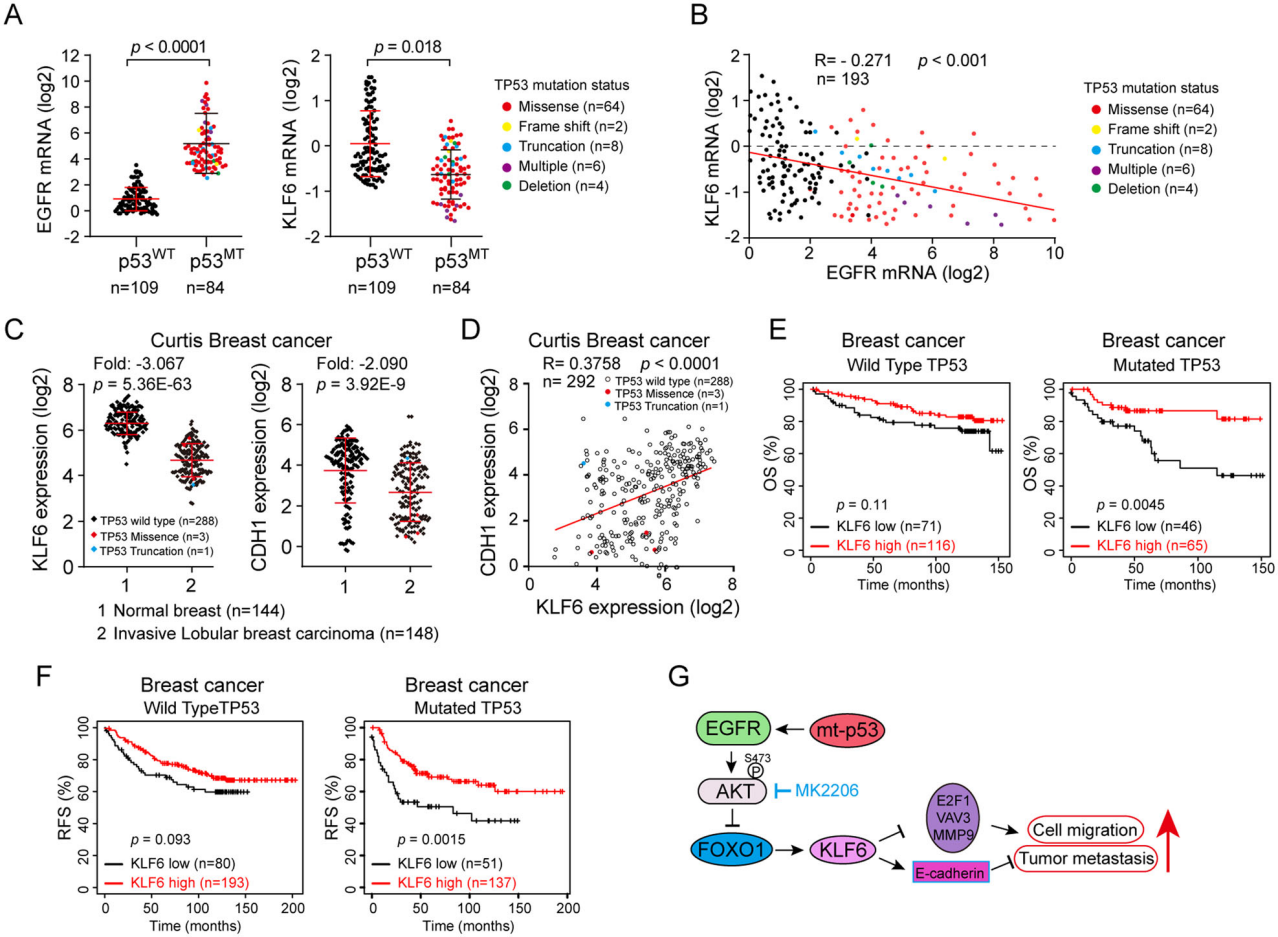
The clinical correlation between expression of EGFR/KLF6 and prognosis of human breast cancer patients bearing WT or mutant p53. **a**, **b** The EGFR and KLF6 mRNA expression levels in human breast cancer biopsy samples with or without p53 mutant were analyzed based on the cBioPortal dataset (TCGA, Cell 2015), the status of mutant p53 marked with a different color (**a**). The same data sets were used for analyses of Pearson correlation coefficient (*R*-value) and a two-tail probability test (*P* value) (**b**). **c**, **d** The KLF6 and CDH1 (encoding E-cadherin protein) mRNA expression levels were analyzed using the Oncomine “Curtis Breast cancer” dataset (**c**). The same data sets were used for analyses of Pearson correlation coefficient (*R*-value) and a two-tail probability test (*P* value) (**d**). **e**, **f** The Kaplan–Meier plots of overall survival (OS) and relapse-free survival (RFS) of human breast cancer patients were stratified by KLF6 mRNA expression levels with or without TP53 mutations, the log-rank test *P* values are shown. **g** A working model illustrates that mutant p53-R273H suppresses KLF6 via EGFR-AKT-FOXO1 in promoting cell migration and tumor metastasis.

We next analyzed the TCGA database for the clinical relevance of KLF6 and EGFR with mutant p53 in human breast cancer specimens. As shown in Fig. 5a, increased EGFR expression or reduced KLF6 expression was significantly correlated with mutant p53 in human breast cancer. Further analyses revealed a significant negative relationship between EGFR and KLF6 (Fig. 5b). In addition, expression of KLF6 and E-cadherin (encoded by CDH1) was significantly downregulated in human invasive breast carcinoma (Fig. 5c) and a significant positive relationship between KLF6 and E-cadherin was observed (Fig. 5d). Notably, low expression of KLF6 was significantly associated with poor OS and RFS in p53 mutated human breast cancer (Fig. 5e, f), but not in p53 wild-type patients.

Taken together, this study demonstrates that mutant p53 represses KLF6–E-cadherin axis via EGFR/AKT/FOXO1 signal pathway to promote cancer cell migration and tumor metastasis (Fig. 5g).

## Discussion

It is well documented that mutant p53 has promoted tumor invasion and metastases. For instance, p53 hotpot mutant proteins can repress E-cadherin expression and consequently induces EMT through modulation of the miR-130b–ZEB1-Snail axis^22^. p53-R175H can also induce EMT and cell migration via activation of Slug^23^ and Twist^24^. In this study, we demonstrate that p53-R273H inhibits expression of KLF6 and E-cadherin to promote cell migration and tumor metastasis. Interestingly, both conformational mutations (R175H) and DNA-contact mutations (R273H and R248QW) can downregulate KLF6 and E-cadherin expression, suggesting that KLF6, like DLX2^19^, is a common denominator of p53 hotspot mutations in promoting tumor metastasis.

It has been reported that mutant p53-R175H can facilitate EGFR recycling, resulting in activation of EGFR signaling to promote invasion^25^. Moreover, p53-R273H can sustain activation of EGFR signaling via suppressing expression of miR-27a, a negative regulator of EGFR mRNA translation^26^. In this study, we show that p53-R273H upregulates EGFR mRNA and protein expression, and consequently activates AKT signaling. In keeping with this finding, clinical analyses show that EGFR expression is significantly increased in p53 mutant human breast cancer samples. However, the precise mechanism with which p53-R273H activates EGFR transcription needs further investigation.

KLF6 is an important tumor suppressor. In this study, we found that p53-R273H inhibits KLF6 expression via EGFR-AKT signaling, adding another layer of cross-talk between two important tumor suppressors, KLF6 and p53. At the molecular levels, p53-R273H activates AKT, resulting in downregulation of FOXO1 protein expression, in keeping with the previous report that activation of AKT promotes FOXO1 proteasomal degradation^27^. Blocking the activation of AKT by MK2206, a phase II inhibitor of AKT for breast cancer patients^28^, can rescue KLF6 expression and suppress p53-R273H-induced cell migration. Importantly, we demonstrate that FOXO1 can restore KLF6 expression which was suppressed by p53-R273H, indicating that FOXO1 mediates p53-R273H-induced suppression of KLF6 and FOXO1 can directly transactivate KLF6, consistent with the previous report^29^.

Notably, in our study, we demonstrate that p53-R273H inhibits KLF6 expression to promote cell migration and tumor metastasis in E-cadherin-dependent or independent fashion, as KLF6 can repress expression of VAV3^15^, E2F1^16^, or MMP9^17^ involved in tumor metastasis (Fig. 5g).

## Materials and methods

### Cell culture and generation of stable cell lines

Human nontransformed mammary epithelial cell MCF-10A, triple-negative breast cancer cell HCC1806 and MDA-MB-468, pancreatic cancer cell MIA PaCa-2, lung cancer cell NCI-H1975 and H1299, and HEK-293T cell lines were obtained from the American Type Culture Collection (ATCC). MCF-10A cells were maintained in DMEM/F-12 media supplemented with 5% horse serum (Invitrogen), 100U penicillin-streptomycin, 10 μg/mL insulin (Sigma), 20 ng/mL epidermal growth factor (Invitrogen), 100 ng/mL cholera toxin (Sigma), and 0.5 μg/mL hydrocortisone (Sigma). HEK-293T, MDA-MB-468, MIA PaCa-2, and H1299 cells were maintained in Dulbecco’s modified Eagle’s medium (DMEM), supplemented with 10% fetal bovine serum (Hyclone Inc, USA). HCC1806 and NCI-H1975 cells were cultured in RPMI 1640, supplemented with 10% fetal bovine serum (Hyclone). Cells were cultured with 1% antibiotic-antimycotic (Gibco, #15240062), at 37 °C in a humidified incubator under 5% CO_2_. Authentication of cells was verified by short tandem repeat DNA profiling.

Lentiviral particles were prepared as described previously^30^. For the generation of stable cell lines, Cells were infected with recombinant lentiviruses. After 48 h, 2 μg/mL puromycin (Sigma Inc, USA) was used to select stably cells expressing desired gene or shRNA.

### Cell morphology assay

Cells were seeded in six-well plate at single cell density and maintained with media at 37 °C in a humidified incubator under 5% CO_2_. After about 7 days, cells were fixed with methanol for 20 min and stained with 0.4% crystal violet for 30 min. A phase-contrast microscope was used to capture cell morphology.

### Transwell assays for cell migration

Cell migration was measured in 24-well plate with 6.5 mm, 8-μm-pore polycarbonate membrane transwell inserts (BD Biosciences). Cells were digested with trypsin and counted. cells were suspended in serum-free media and seeded into the inner chamber (5 × 10^4^). The outer chamber was filled with normal growth media. After 12 h, serum-free media were removed, cells were washed with PBS, fixed with methanol for 20 min and stained with 0.4% crystal violet for 30 min. Non-migrating cells in inner chamber were removed with a cotton swab. Migrating cells were photographed with microscope in five random fields and counted with Image J software.

### In vivo metastasis assays

The sample size for animal studies was designed according to previous report^31^. BALB/c nude female mice (6 weeks old, DaShuo Biotechnology, ChengDu, China) were randomly divided into three groups (ten mice per group) and housed under standard conditions. H1299 stable cells (1 × 10^6^) in 150 μL saline were injected into the lateral tail vein of mice. Mice were sacrificed and dissected after 50 days. Lungs were extracted, inspected metastatic nodules with a dissecting microscope, fixed, embedded, and sectioned. Lung sections were stained with hematoxylin and eosin (H&E), and photographed with microscope. The numbers of metastatic lesion were counted according to the photograph and no blinding test was used in assessing the outcome. All animal experiments in this study were approved by the Institutional Animal Care and Use Committee (IACUC) of Sichuan University, and the procedures were performed according to the guidelines established by the China Council on Animal Care.

### Plasmid construction

Human p53-R273H, p53-R175H, or p53-R248W plasmids as kind gifts from Dr. Bert Vogelstein. Human KLF6, FOXO1, or E-Cadherin genes were sub-cloned into pLVX-Puro vector. KLF6 mutants, including A123D, L169P, L217S, and C265Y, were generated by the KOD-Plus-Mutagenesis kit (SMK-101, Toyobo Osaka). shRNA oligos were cloned into pLKO.1 vector according to protocol from Addgene instruction. Shp53, shKLF6, and shEGFR oligos sequence are listed below: shp53-#1: 5′-GAGGGATGTTTGGGAGATGTA-3′, shp53-#2: 5′-CACCATCCACTACAACTACAT-3′; shKLF6-#1: 5′-ACTCAGATGTCAGCAGCGAAT-3′, shKLF6-#2: 5′-GCTCCCACTGTGACAGGTGTT-3′; shEGFR: 5′-CGCAAAGTGTGTAACGGAATA -3′. All plasmids in this study were confirmed through DNA sequencing.

### Western blot analyses

Cells were lysed in EBC250 lysis buffer (250 mM NaCl, 25 mM Tris, pH 7.4, 0.5% Nonidet P-40, 50 mM NaF, 0.5 mM Na3VO4, 0.2 mM phenylmethylsulfonyl fluoride, 20 μg/mL aprotinin, and 10 μg/mL leupeptin). Equal amounts of total proteins were separated by SDS-PAGE, transferred to PVDF membrane and hybridized to an appropriate primary antibody and HRP-conjugated secondary antibody for subsequent detection by enhanced chemiluminescence.

Antibodies used in this study were as follows: p53 (DO-1, 1:2000, Code No. sc-126, Santa Cruz, CA, US), KLF6 (1:1000, Code No. 14716-1-AP, Proteintech, IL, US), N-cadherin (1:1000, Code No. 2447-1, Abcam, Cambridge, MA, US), Vimentin (1:1000, Code No. sc-7557, Santa Cruz), E-Cadherin (1:1000, Code No. 40772, Abcam), GAPDH (1:3000, Code No. EM1101, HuaBio, Hangzhou, China), p-AKT (1:1000, Code No. 4058, Cell Signaling Technology (CST), MA, US), AKT (1:1000, Code No. 9272, CST), FOXO1(1:1000, Code No. 2880, CST), EGFR (1:1000, Code No. 2232, CST), and p21(1:1000, No. 2947, CST).

### Quantitative RT-PCR

Total RNAs from target cells were extracted using the RNA extraction kit (Qiagen, Germany) according to the manufacturer’s instruction. For cDNA synthesis, total RNAs were performed reverse transcription using ReverTra Ace qPCR RT Master Mix (FSQ-201, Toyobo, Japan). SYBR Green Master Mix reagent (Bio-Rad) and other reactants were carried out at 95 °C for 30 s, followed by 40 cycles of 95 °C for 5 s and 65 °C for 15 s in CFX96 Real-Time System (Bio-Rad). GAPDH was used as an endogenous reference gene to normalize target gene expression by the ΔΔCt method. qPCR primer sequences are listed below: GAPDH-F: 5′-TGGACTCCACGACGTACTCA-3′, GAPDH-R: 5′-AATCCCATCACCATCTTCCA-3′; CDH1-F: 5′-GGATGTGCTGGATGTGAATG-3′, CDH1-R: 5′-CACATCAGACAGGATCAGCAGAA3′: KLF6-F: 5′-GGCAACAGACCTGCCTAGAG-3′, KLF6-R: 5′-CTCCCGAGCCAGAATGATTTT-3′; EGFR-F: 5′-AAGTGTAAGAAGTGCGAAGG-3′, EGFR-R: 5′-GGAGGAGTATGTGTGAAGGA-3′.

### Integrated analyses of gene expression profiles and ChIP-seq data

The effects of p53-R273 on gene expression was analyzed by RNA-Seq, as described^19^. ChIP-seq data were derived from a previous report, in which U251 cells harboring p53-R273H were used for ChIP-Seq analyses using a p53-specific antibody^20^. Integrative analysis of the differentially expressed genes (fold-change cutoff of 1.2) from the RNA-Seq data with the ChIP-Seq identified genes were obtained.

### Human data from publicly available data sets

TCGA data sets (cBioPortal, www.cbioportal.org/) were used to analyze breast carcinoma (TCGA, Cell 2015) for EGFR and KLF6 expression in p53 mutation samples. The Curtis Breast dataset from Oncomine database (https://www.Oncomine.org/resource/login.html) was used for analyses of KLF6 or CDH1 gene expression. Kaplan–Meier survival graphs were generated from data available from KM Plotter (www.kmplotter.com)^32^ by using the probe “208960_s_at”.

### Statistical analyses

Data from cell culture were performed in three independent experiments. Data were presented as means ± SD. The differences between two groups were performed using the two-tailed unpaired Student’s *t* test. The differences between clinical data were compared after homogeneity tests. *p* values < 0.05 were considered statistically significant.
